# Parkinsonian symptoms in normal pressure hydrocephalus: a population-based study

**DOI:** 10.1007/s00415-017-8598-5

**Published:** 2017-09-06

**Authors:** Karin Molde, Lars Söderström, Katarina Laurell

**Affiliations:** Department of Pharmacology and Clinical Neuroscience, Umeå University, Umeå, Sweden

**Keywords:** Normal pressure hydrocephalus, Hydrocephalus, Parkinsonism, Parkinson’s disease, UPDRS

## Abstract

It may be challenging to differentiate normal pressure hydrocephalus (NPH) from neurodegenerative disorders such as Parkinson’s disease. In this population-based study, we wanted to describe the frequency of parkinsonian symptoms among individuals with and without NPH, and whether the motor examination part of the Unified Parkinson’s Disease Rating Scale (UPDRS-m) score differs between these groups. Furthermore, we wanted to find out whether there was a relationship between UPDRS-m score, NPH symptoms, and radiological signs of NPH. A sample of 168 individuals over the age of 65 with and without self-reported symptoms of NPH underwent a computerized tomography of the brain and clinical examination, including UPDRS-m to grade parkinsonian symptoms. According to diagnostic guidelines, 38 fulfilled criteria for NPH, whereas 130 had unlikely NPH. Bradykinesia was significantly more common among those with NPH (79%) compared to those with unlikely NPH (32%) (*p* < 0.001). The corresponding figures for rigidity were 43 vs. 15% (*p* < 0.001), for postural instability 71 vs. 22% (*p* < 0.001), and for tremor at rest 5 vs. 6% (not significant). The total UPDRS-m score was significantly higher among individuals with NPH (median = 12) than without (median = 1) and correlated significantly with the degree of NPH symptoms (*r* = −0.72) and ventriculomegaly (*r* = 0.31). In this study, parkinsonian symptoms, except resting tremor, were frequent in individuals with NPH and correlated with the severity of NPH symptoms. Asymmetric symptoms were uncommon. We recommend a liberal use of neuroradiological imaging when investigating a patient with parkinsonian features.

## Introduction

Parkinsonian symptomatology exists in addition to the classical triad of gait, cognitive, and urinary symptoms in normal pressure hydrocephalus (NPH) [1–4], and may complicate the diagnostic considerations [5, 6]. Radiology of the brain is essential to diagnose NPH [7], with typical findings of dilated ventricles without any macroscopic obstruction to cerebral spinal fluid (CSF) flow, often with signs of compressed cortical sulci combined with focally enlarged sulci [8]. NPH is a treatable condition; in about 80% of the patients, the symptoms improve after surgical treatment with CSF shunt [9].

The Unified Parkinson’s Disease Rating Scale (UPDRS) [10] is a widely used rating scale for Parkinson’s disease (PD) [11] and the motor examination part (UPDRS-m) has also been used to rate parkinsonian motor symptoms in NPH patients [12–15]. In the previous hospital-based studies, upper body bradykinesia has been described in 62% and parkinsonism in up to 71% of NPH patients [2, 12]. Significant improvements in the total UPDRS-m score have been described after shunt surgery and after CSF removal by lumbar puncture (CSF tap test) [12–15]. Mild parkinsonian signs [16] in the four categories bradykinesia, tremor at rest, rigidity, and postural/gait changes are found in 20–40% of the older population [17, 18]. Except from neurodegenerative diseases, factors associated with normal aging as well as comorbidities such as cerebrovascular disease and essential tremor may contribute to findings of isolated parkinsonian signs [16, 19].

As parkinsonian features are important in the differential diagnosis of neurological disorders, we wanted to describe the frequency of such symptoms and compare the UPDRS-m score between individuals from the general population with and without signs of NPH. Furthermore, we wanted to find out whether there was a relationship between UPDRS-m score, NPH symptoms, and radiological signs of NPH.

## Methods

### Material

**Fig. 1:**
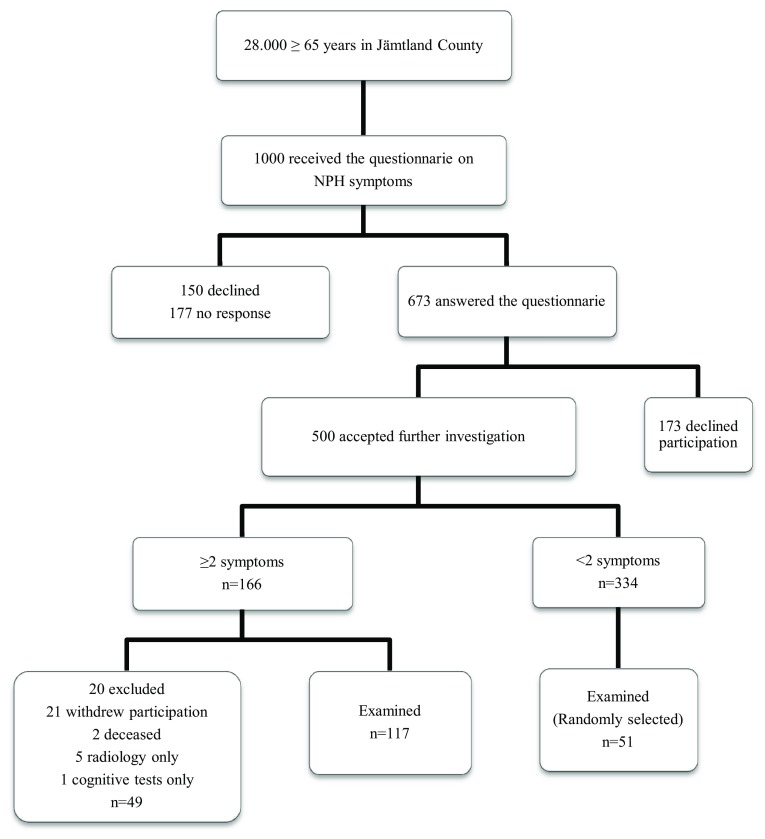
Flow chart of sample selection. Final study population, *n* = 168

This study is part of an ongoing epidemiological study on the prevalence of NPH. Out of the total population of 28,000 individuals aged 65 years or older living in Jämtland County, 1000 were randomly selected from the Swedish population register and received a questionnaire on NPH symptoms. The questionnaire is based on an on-line screening tool for NPH [20], and consists of seven yes or no questions regarding balance and gait disturbance, cognitive impairment, and urinary symptoms. Individuals who reported two symptoms (including gait or balance disturbance) or more were invited to undergo further investigations. In total, 673 returned a correct filled in questionnaire giving a response rate of 67.3%, of which 168 individuals with and without symptoms of NPH underwent computerized tomography (CT) of the brain and neurological examinations. The flow chart describes the selection of the final study population (Fig. 1). Exclusion criteria were severe medical conditions sufficient to explain the symptoms, for example known brain tumor or severe multiple sclerosis diagnosed by a neurologist. Among those who returned the questionnaire and accepted further studies, one reported that he was under treatment for idiopathic PD at the Neurology Department. He had no signs of NPH on a previous CT scan and was excluded from further studies. Among the investigated 168 individuals, two had tested dopaminergic treatment but discontinued because of lack of effect. One of them had an ischemic lesion in basal ganglia on CT brain, whereas the other had radiological signs of NPH and received a shunt in June 2016 with a clear improvement.

To diagnose NPH, we used the guidelines by Mori et al. [7], which are suitable for population-based studies, because they can be used without CSF pressure measurement. The criteria for “possible NPH” require at least two symptoms from the clinical triad; gait disturbance, cognitive impairment, and urinary symptoms. Ventricular dilation (Evans’ index> 0.3) is mandatory. “Probable NPH” requires in addition to the criteria for “possible NPH”, a CSF pressure of 200 mmH_2_O, or less. However, when CSF pressure measurements are not performed, as in the present study, the diagnosis “possible NPH with neuroradiological support” can be used instead. It requires an NPH-specific radiological picture of the brain with narrowing of the sulci and subarachnoid spaces over the high convexity/midline surface [21]. Finally, the diagnosis “definitive NPH” is used when the symptoms improve after shunt surgery. To simplify, we denominated the groups as “unlikely”, “possible”, and “probable” NPH where the latter was equivalent to “possible NPH with neuroradiological support”. The only two individuals with the diagnosis of “definite NPH”, i.e., confirmed with shunt surgery were included in the “probable NPH” group.

**Fig. 2:**
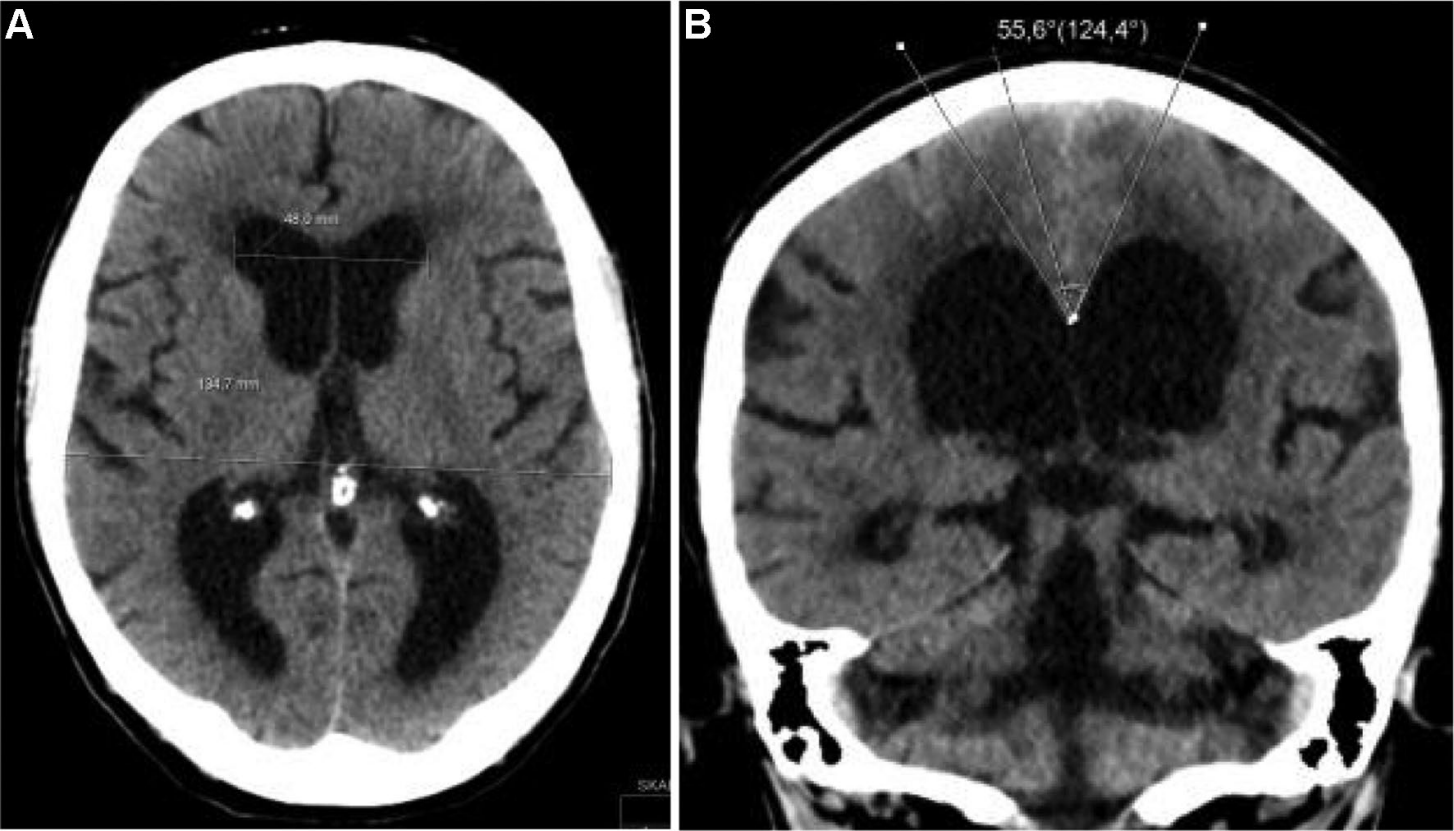
Radiological markers of NPH shown on CT scan **a** Evans’ index (ratio of maximum width of the frontal horns to the maximum inner skull diameter) >0.3; **b** callosal angle <60°, narrow medial sulci, dilated fissure Sylvii, and dilated temporal horns

Radiological evaluation was made with a CT scan of the brain. Previously described radiological markers were analyzed, i.e., Evans’ index, callosal angle, signs of narrow medial sulci, focally enlarged sulci, dilated fissure Sylvii, and size of temporal horns [8] (Fig. 2).

To investigate the degree of parkinsonian motor symptoms, UPDRS-m [10] was used, grading the patients motor function from 0 to 4, where 0 represents normal and 4 represents severe impairment. Tremor, rigidity, and bradykinesia in extremities are measured and scored bilaterally. A senior consultant in neurology with assistance of a trained medical student made all the examinations. Tremor at rest, bradykinesia in extremities, rigidity, and postural instability were described as present when scored 1 or more. Parkinsonism was defined according to the UK brain bank criteria, as the presence of bradykinesia together with at least one of the symptoms tremor at rest, rigidity, and postural instability [22].

We used a scale developed by Hellström et al. [23] to measure the severity of NPH symptoms. The scale is composed of four different domain scores (gait, balance, neuropsychology, and continence) which are assessed by 10 m walking test, ordinal ratings of gait and balance, Grooved pegboard test, Ray Auditory Verbal Learning Test (RAVLT), Stroop test, and an ordinal continence scale based on self-reports [23]. A total NPH score is calculated as the mean of the four domain scores with the gait domain counted twice. A score of 100 means the absence of symptoms, and 0 is the most severe state.

The neurologist was blinded to radiological data and, accordingly, the radiologist was blinded to clinical data.

### Statistical analysis

Descriptive statistics were used to present the frequency of different parkinsonian symptoms among individuals with and without signs of NPH.

Differences in the level of parkinsonian symptoms and UPDRS score between the three groups, “unlikely”, “possible”, and “probable” NPH, were tested with the Fisher–Freeman–Halton exact test and Kruskal–Wallis statistical test, respectively.

Spearman correlation analyses were used to measure the association between UPDRS-m score and NPH score and the UPDRS-m score and continuous radiological variables (i.e., Evans’ index, callosal angle, and size of temporal horns), respectively. The level of significance was set to *p* < 0.05.

All analyses were performed using SPSS (IBM SPSS Statistics for Macintosh, Version 24.0, IBM Corp.).

### Ethical approval

The Regional Ethical Review Board in Umeå approved the study (Dnr 2014/180-31) and all participants gave written, informed consent.

## Results

The sample consisted of 168 individuals, 75 men and 93 women, mean 75 (66–92) years. According to diagnostic criteria, 11 individuals had “probable NPH”, 27 individuals had “possible NPH”, and 130 had “unlikely NPH”. Table 1 shows the demography of the study population.

**Table 1:** Demography of the study population

	Unlikely NPH (*n* = 130)	Possible NPH (*n* = 27)	Probable NPH (*n* = 11)
*n* male (%)	51 (39)	16 (59)	8 (73)
Mean age (SD)	74 (5.87)	79 (7.65)	80 (7.29)

*NPH* normal pressure hydrocephalus

The frequency of specific parkinsonian symptoms according to NPH diagnosis is shown in Table 2. Among those with NPH (i.e., “possible NPH” and “probable NPH”), bradykinesia was found in 79%, of which in upper extremities in 68%. The corresponding figures for tremor at rest were 5%, rigidity 43%, and postural instability 71%, respectively. Parkinsonism was found in 71% of those with NPH, and in 20% of those with unlikely NPH.

**Table 2:** Frequency of parkinsonian symptoms according to diagnosis

	Unlikely NPH (*n* = 130) *n* (%)	Possible NPH (*n* = 27) *n* (%)	Probable NPH (*n* = 11) *n* (%)	*p* value
Bradykinesia in extremities (*n* = 167)	41 (32)	20 (74)	10 (91)	<0.001
Unilateral/bilateral	16 (12)/25 (20)	7 (26)/13 (48)	0 (0)/10 (91)	
Only arm/hand	28 (22)	10 (37)	4 (36)	
Only leg	3 (2)	3 (11)	1 (9)	
Tremor at rest	8 (6)	1 (4)	1 (9)	0.690
Unilateral/bilateral	3 (2)/1 (1)	0 (0)/1 (4)	1 (9)/0 (0)	
Only arm	4 (3)	0 (0)	1 (9)	
Only leg	0 (0)	0 (0)	0 (0)	
Head/face tremor	7 (5)	0 (0)	0 (0)	
Rigidity (*n* = 162)	19 (15)	6 (25)	9 (82)	<0.001
Unilateral/bilateral	5 (4)/14 (11)	2 (8)/4 (17)	4 (36)/5 (46)	
Only arm	8 (6)	2 (8)	1 (9)	
Only leg	4 (3)	0 (0)	0 (0)	
Postural instability	29 (22)	18 (67)	9 (82)	<0.001

*NPH* normal pressure hydrocephalus

Except for facial expression and tremor, the UPDRS-m scores differed significantly between the groups of “unlikely”, “possible”, and “probable” NPH (Table 3). As expected, the difference was most marked between individuals with “unlikely” and “probable” NPH, and further pronounced for the total UPDRS-m score, where those without NPH had a median score of 1 (0–23) and those with NPH (i.e., “possible” and “probable” NPH) had a median score of 12 (0–35).

**Table 3:** Differences in UPDRS-m score between “unlikely”, “possible”, and “probable” NPH

	Unlikely NPH (*n* = 130)		Possible NPH (*n* = 27)		Probable NPH (*n* = 11)		*p* value
	Mean ± SD	Median (range)	Mean ± SD	Median (range)	Mean ± SD	Median (range)	
Speech	0.02 ± 0.20	0 (0–2)	0.00 ± 0.00	0 (0–0)	0.27 ± 0.65	0 (0–2)	0.002
Facial expression	0.05 ± 0.23	0 (0–1)	0.11 ± 0.32	0 (0–1)	0.18 ± 0.41	0 (0–1)	0.197
Tremor at rest^a^	0.09 ± 0.40	0 (0–3)	0.15 ± 0.77	0 (0–4)	0.09 ± 0.30	0 (0–1)	0.829
Action or postural tremor^b^	0.39 ± 1.11	0 (0–6)	0.85 ± 1.59	0 (0–6)	0.18 ± 0.40	0 (0–1)	0.094
Rigidity (*n* = 162)^c^	0.42 ± 1.29	0 (0–8)	0.83 ± 1.74	0 (0–6)	2.82 ± 2.32	2 (0–7)	<0.001
Finger taps^b^	0.32 ± 0.77	0 (0–4)	0.93 ± 1.14	0 (0–4)	1.09 ± 1.04	1 (0–3)	<0.001
Hand movements^b^	0.33 ± 0.77	0 (0–4)	0.81 ± 1.04	0 (0–3)	1.18 ± 0.87	1 (0–2)	<0.001
Rapid alternating movements^b^	0.37 ± 0.86	0 (0–4)	1.11 ± 1.43	0 (0–4)	1.82 ± 1.08	2 (0–3)	<0.001
Leg agility^b^	0.18 ± 0.57	0 (0–3)	0.67 ± 1.04	0 (0–4)	1.27 ± 1.42	1 (0–4)	<0.001
Rising from chair (*n* = 167)	0.26 ± 0.63	0 (0–3)	0.67 ± 0.88	0 (0–3)	1.00 ± 0.89	1 (0–2)	<0.001
Posture (*n* = 167)	0.27 ± 0.51	0 (0–3)	0.52 ± 0.64	0 (0–2)	1.09 ± 0.94	1 (0–3)	<0.001
Postural stability	0.29 ± 0.60	0 (0–3)	1.00 ± 1.00	1 (0–4)	1.36 ± 1.12	1 (0–4)	<0.001
Gait	0.43 ± 0.81	0 (0–3)	1.04 ± 0.76	1 (0–3)	1.82 ± 0.87	2 (1–3)	<0.001
Body bradykinesia (*n* = 167)	0.22 ± 0.50	0 (0–2)	0.93 ± 0.73	1 (0–2)	1.64 ± 0.81	2 (0–3)	<0.001
Total score, max = 108 (*n* = 162)	3.55 ± 5.41	1 (0–23)	9.00 ± 6.87	8 (0–25)	15.82 ± 8.35	15 (3–35)	<0.001

*UPDRS-m* Unified Parkinson’s Disease Rating Scale-motor examination part, *NPH* normal pressure hydrocephalus

^a^Total score face + arms + legs

^b^Items measured bilaterally show score right + left

^c^Total score neck + arms + legs

**Fig. 3:**
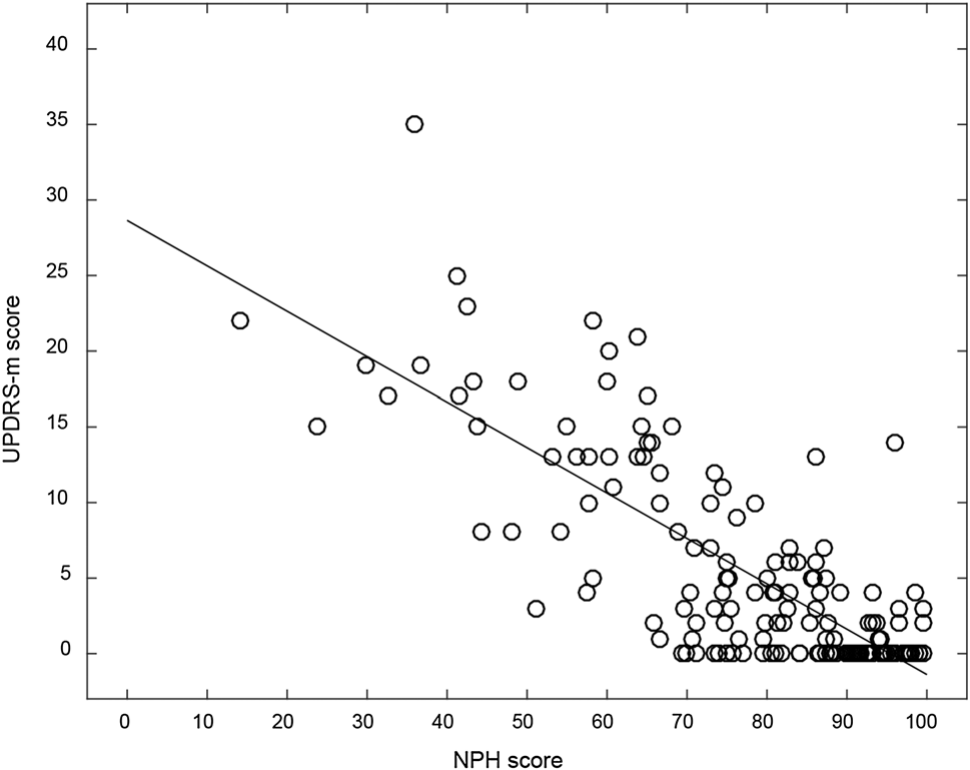
Scatterplot, illustrating the association between the NPH score (*x*-axis) and UPDRS-m score (*y*-axis) among the 168 study participants, *r* = −0.72, *p* < 0.001

The score of NPH symptoms [23] correlated significantly with score on the UPDRS-m (*r* = −0.72, *p* < 0.001) (Fig. 3). Furthermore, the radiological markers Evans’ index and size of temporal horns correlated significantly with the UPDRS-m score (*r* = 0.31 and *r* = 0.39, *p* < 0.001), whereas the radiological marker callosal angle (*r* = −0.11) did not.

## Discussion

In this population-based study, bradykinesia and rigidity were more than twice, and postural instability more than three times as frequent in individuals with NPH than in those without NPH. In contrast, tremor at rest was unusual and similar between the two groups. The total UPDRS-m score was significantly higher among individuals with NPH and correlated to the severity of NPH symptoms. Our findings confirm the results from the previous studies on hospital-based material that parkinsonism can be a part of the clinical syndrome of NPH [2, 3, 12–15].

Of the 38 individuals with NPH, 71% showed parkinsonism according to the UK Brain Bank Criteria [22]. This equals the frequency of parkinsonism (71%) reported in a study of 17 shunt-responsive (definitive) NPH patients [12], where the definition of parkinsonism was the presence of at least two symptoms out of bradykinesia, tremor at rest, rigidity, or postural instability. With the same definition, the frequency of parkinsonism would only be slightly higher (74%) in our material. In our NPH sample, upper body bradykinesia was slightly higher (68 vs. 62%) and rigidity three times as prevalent (43 vs. 14%) compared to a hospital-based study of 65 NPH patients who improved after CSF removal and were considered for surgery [2]. This might be explained by that the cut-off level for bradykinesia was lower in our study (UPDRS-m score = 1) than in their study (UPDRS-m score = 2), and that we, in contrast to them, included paratonia in the assessment of rigidity. However, one also has to consider a selection bias in clinical material in that patients with parkinsonian features might be less often considered for shunt surgery.

Parkinsonian signs were also present in the group with “unlikely NPH” where we found bradykinesia in one-third of the individuals evaluated, rigidity in one out of seven, and postural instability in one out of five. These results are similar to those found in the previous population-based studies [17, 18].

The pathophysiology of NPH is not fully understood. Theories involve a high resistance to CSF outflow contributing to ventricular enlargement, mechanical pressure of the brain parenchyma, disturbance of cerebral blood flow (CBF), and increased water content in periventricular areas [24, 25]. In NPH patients, significantly reduced CBF in the thalamus, the head of caudate nucleus and putamen has been shown with positron emission tomography (PET) [26]. A study with fluorodopa PET in a patient with obstructive hydrocephalus who developed parkinsonism due to shunt dysfunction revealed reduced uptake in the caudate and putamen [27]. Further indications of a disturbance in the nigrostriatal pathway were provided in a recent study of 30 patients with NPH and parkinsonism where striatal dopaminergic deficit on dopamine transporter (DaT) scan was found in almost half of the patients [14]. Whether such disturbances are due to global or regionally reduced CBF [25, 26, 28–30] or other mechanisms is not fully clear. A reduction in postsynaptic D2 receptors binding in the putamen of NPH patients has been demonstrated [31], and there are theories of changes in additional dopaminergic pathways as well that also contribute to parkinsonism in NPH [2, 12, 32].

The reversibility of parkinsonian signs that has been reported after shunt surgery or CSF removal supports that these symptoms are caused by hydrocephalus and not just a result of comorbidity [12–15]. A recent hospital-based study with 55 NPH patients who underwent a CSF tap test revealed a significant improvement of bradykinesia in upper and lower bodies, whereas tremor marginally improved and rigidity did not improve [15]. In a recent publication, oral dopaminergic therapy added a positive effect to shunt surgery in the improvement of the UPDRS-m score in patients with NPH and parkinsonism, indicating that the disturbance in the dopaminergic system might not be totally reversible [14].

The findings in the present study highlight some of the diagnostic challenges when meeting a patient with parkinsonian features. However, there are some clinical hallmarks that can be useful. In contrast to PD [22], tremor at rest seems uncommon among individuals with NPH [2], and according to our results, asymmetric symptomatology is less common as well. In PD, non-motor symptoms such as olfactory dysfunction and rapid eye movement (REM) sleep behavior disorder are common and can precede the motor symptoms [33], and such symptoms would be valuable to study also in NPH patients to see if their presence could help in differentiating the disorders from each other [34]. Neuropsychiatric symptoms as depression and anxiety are frequent in both conditions [35]. When comparing the gait disturbance in PD and NPH, Stolze et al. [32] found reduced velocity and stride length, freezing phenomenon, and reduced cadence in both groups; in addition, NPH patients had a broad-based gait, outwardly rotated feet, and a diminished step height. Nevertheless, we agree with Bugalho et al. [36] that it might be difficult to differ PD from NPH only by gait function. Bradykinesia of the hand is showed to share the same features in NPH patients as in patients with PD [37], and even experienced neurologists might mistake these disorders [5, 6]. In addition, the disorders may co-occur. These circumstances illustrate the importance of a liberal use of radiological investigation when the PD symptoms are atypical or do not respond to dopaminergic treatment.

Although it may complicate the diagnostic procedure, parkinsonian symptomatology in NPH should not exclude the patient from shunt surgery, as the symptoms diminish postoperatively [12–14].

The strength of this study is the relatively large, unselected sample with individuals from the general population. Only three individuals had been under investigation for NPH: one had received a shunt a few years prior to the study, one received a shunt afterwards (June 2016), and one declined operation. The shunt operated patients improved postoperatively; they were both wheelchair bound before the operation and regained walking ability.

The study has some limitations, as well. We did not exclude individuals with common comorbidities such as arthrosis and vascular disease, to minimize the risk of excluding those who suffered from NPH as well. Although this might have influenced the UPDRS-m score, we believe that it increases the generalizability of the results. This is supported by the fact that the group without NPH did not show more parkinsonian symptoms than what has been previously reported in the general population of elderly [17, 18]. We used CT scans as the neuroradiological evaluation instead of MRI which is suggested in the diagnostic guidelines [7], but this should be of minor importance as most radiological signs of NPH are seen on a CT scan of the brain as well. Likewise, we did not assess the lumbar opening pressure which, according to guidelines, should not exceed 200 mmH_2_O in “probable NPH” [7]. However, according to clinical experience, a pressure slightly above this level is not uncommon among NPH patients, and to rule out a non-communicating hydrocephalus, with clearly increased CSF pressure, neuroradiological imaging should be used instead.

Finally, we do not yet know how many of those with NPH in this study will be offered and respond to shunt surgery, i.e., fulfill the diagnosis of “definitive NPH”.

## Conclusion

In this study, parkinsonian motor symptoms, except resting tremor, were frequent among individuals with NPH and correlated with the severity of NPH symptoms. Asymmetric distribution was rare. We recommend a liberal use of neuroradiological imaging when investigating a patient with parkinsonian features, in particular when the symptoms do not response to dopaminergic treatment.
